# Low Brachial Artery Flow-Mediated Dilation Predicts Worse Prognosis in Hospitalized Patients with COVID-19

**DOI:** 10.3390/jcm10225456

**Published:** 2021-11-22

**Authors:** Vanessa Bianconi, Massimo Raffaele Mannarino, Filippo Figorilli, Elisabetta Schiaroli, Elena Cosentini, Giuseppe Batori, Ettore Marini, Amirhossein Sahebkar, Francesco Grignani, Anna Gidari, Daniela Francisci, Matteo Pirro

**Affiliations:** 1 Unit of Internal Medicine, Department of Medicine and Surgery, University of Perugia, 06129 Perugia, Italy; vanessa.bianconi@ospedale.perugia.it (V.B.); filippofigorilli@gmail.com (F.F.); elena.cosentini@libero.it (E.C.); giusbatori92@gmail.com (G.B.); et.marini@gmail.com (E.M.); francesco.grignani@unipg.it (F.G.); matteo.pirro@unipg.it (M.P.); 2 Unit of Infectious Diseases, Department of Medicine and Surgery, University of Perugia, 06129 Perugia, Italy; elisabetta.schiaroli@unipg.it (E.S.); annagidari91@gmail.com (A.G.); daniela.francisci@unipg.it (D.F.); 3 Biotechnology Research Center, Pharmaceutical Technology Institute, Mashhad University of Medical Sciences, Mashhad 1696700, Iran; amir_saheb2000@yahoo.com; 4 Neurogenic Inflammation Research Center, Mashhad University of Medical Sciences, Mashhad 1696700, Iran; 5 School of Pharmacy, Mashhad University of Medical Sciences, Mashhad 1696700, Iran

**Keywords:** SARS-CoV-2, COVID-19, pneumonia, endothelial dysfunction, bFMD

## Abstract

Background: Endothelial injury can be induced by coronavirus disease 2019 (COVID-19) and seems to exert a crucial pathogenic role in its most severe clinical manifestations. We aimed to investigate the association between brachial artery flow-mediated dilation (bFMD), a potential clinical and non-invasive measure of endothelial function, and in-hospital prognosis of COVID-19 patients. Methods: Brachial artery flow-mediated dilation was assessed in hospitalized COVID-19 patients within 48 h of hospital admission. The association between bFMD and either intensive care unit (ICU) admission or in-hospital death was explored using univariable and multivariable analyses. Results: Four hundred and eight patients were enrolled. Significantly lower bFMD values emerged in COVID-19 patients with either radiographic signs of pneumonia, respiratory distress, or the need for non-invasive ventilation compared with patients without these signs (*p* < 0.001, *p* = 0.001, and *p* < 0.001, respectively). Forty-two (10%) patients were admitted to the ICU, 76 (19%) patients died, and 118 (29%) patients met the composite endpoint of ICU admission/in-hospital death. At unadjusted Cox regression analysis showed that low bFMD (<4.4%, the median value) was associated with a higher risk for the composite endpoint of ICU admission/in-hospital death compared with high bFMD (≥4.4%, the median value) (HR 1.675, 95% CI 1.155–2.428, *p* = 0.007). Multi-adjusted Cox regression analyses showed that low bFMD was independently associated with a 1.519- to 1.658-fold increased risk for the composite endpoint of ICU admission/in-hospital death. Conclusions: Low bFMD predicts an unfavorable in-hospital prognosis in COVID-19 patients. The measurement of bFMD may be clinically useful in the prognostic stratification of COVID-19 patients upon hospital admission.

## 1. Introduction

The coronavirus disease 2019 (COVID-19) pandemic caused by severe acute respiratory syndrome coronavirus 2 (SARS-CoV-2) continues to be a dramatic healthcare emergency worldwide, reaching a total of 232,075,351 confirmed cases and 4,752,988 deaths as of 27 September 2021 [1,2]. Although trends toward a decrease in in-hospital mortality rates have been reported over time since the outbreak of the pandemic, elevated numbers of daily COVID-19-related deaths continue to be observed in the hospital setting, especially in countries in which vaccination campaigns are progressing more slowly [3,4]. Undoubtedly, the unavailability of effective therapeutic strategies, which causes the clinical management to be mainly based on supportive measures, is implicated in the high rates of unfavorable outcomes in hospitalized COVID-19 patients [5,6,7]. Therefore, patient inclusion in ongoing clinical trials and investigational off-label uses of drugs are currently employed in clinical practice as additional therapeutic options beyond those recommended by available guidelines [8]. In this critical scenario, the stratification of COVID-19 severity and prognosis at hospital admission may be useful to tailor the intensity and choice of therapeutic strategies [9].

The endothelium is profoundly injured during SARS-CoV-2 infection because of the direct cytopathic effect of the virus and the overactivation of the systemic inflammatory response [10]. Particularly, SARS-CoV-2 can infect endothelial cells and replicate within them [10,11]. In addition, the cytokine storm elicited by viral infection has been found to induce endothelial activation (i.e., the increased expression of adhesion molecules favoring the recruitment of inflammatory cells) and promote endothelial cell apoptosis, thereby leading to the disruption of the endothelial barrier between the blood vessels and the tissues [10,11,12,13]. In addition, compelling evidence suggests that by promoting inflammation and microvascular thrombosis, endothelial damage and dysfunction can impair organ perfusion and promote organ damage, thereby exerting a crucial pathogenic role in the onset of the most severe clinical manifestations of COVID-19, which can be pulmonary or extra-pulmonary [10].

In the light of these lines of evidence, great interest has arisen in the search for markers of endothelial injury with potential clinical utility for the stratification of COVID-19 prognosis [12,14]. To date, several cross-sectional, retrospective, and prospective studies have reported significant discriminatory value of some laboratory markers of endothelial dysfunction and damage (e.g., circulating endothelial cells, soluble Intercellular Adhesion Molecule 1, and von Willebrand Factor Antigen) toward COVID-19 severity and clinical outcomes [15,16,17,18]. However, to the best of our knowledge, no data are yet available on the potential association between the clinical parameters of endothelial dysfunction and in-hospital outcomes of COVID-19.

The aim of this study was to investigate the role of brachial artery flow-mediated dilation (bFMD), a potential clinical and non-invasive measure of endothelial function, in the prediction of the composite endpoint of intensive care unit (ICU) admission/in-hospital death in hospitalized COVID-19 patients.

## 2. Materials and Methods

### 2.1. Study Population

Hospitalized COVID-19 patients referred to the Internal Medicine and Infectious Diseases wards of Santa Maria della Misericordia Hospital of Perugia (Italy) from December 2020 to May 2021 were consecutively enrolled. The study protocol was developed in accordance with the principles of the Helsinki Declaration and was approved by the local ethics committee. The inclusion criteria were as follows: (1) age ≥18 years, (2) a positive result on real-time reverse transcriptase PCR (RT-PCR) assay testing for SARS-CoV-2 on nasal or pharyngeal swab specimens at hospital admission, and (3) informed written consent. The technical impossibility of carrying out the bFMD procedure due to bilateral ankylosis of the elbow, brachial peripheral venous accesses, and/or orthopedic plaster casts were the exclusion criteria. As no previous literature data on the association between bFMD and in-hospital outcomes of COVID-19 patients were available at the time of study initiation, the study sample size was arbitrarily set.

### 2.2. Data Collection

For each patient, data on demographic characteristics, coexisting medical conditions, current treatments, laboratory tests, and physical and instrumental examinations performed at hospital admission were collected and registered in the medical records. Tests for SARS-CoV-2 on nasal or pharyngeal swab specimens were performed through RT-PCR assays (Allplex 2019-nCoV Assay, Seegene, Seoul, South Korea or Xpert Xpress SARS-CoV-2, Cepheid, Sunnyvale, CA, USA). Arterial and venous blood samples were processed according to standard laboratory techniques in order to determine the following laboratory variables: blood gas parameters (ABL90 FLEX blood gas analyzer, Radiometer, Brønshøj, Denmark), leukocyte and platelet count (Sysmex XT-2000i, Dasit, Milano, Italy), D-dimer (BCS XP Coagulation Analyzer, Siemens, Munich, Germany), high-sensitivity cardiac troponin (hs-cTn) (UniCel DxI 800 analyzer, Beckman Coulter, Brea, CA, USA), C-reactive protein (CRP), blood urea nitrogen (BUN), creatinine, and lactate dehydrogenase (LDH) (AU5800 Clinical Chemistry System, Beckman Coulter, Brea, CA, USA). The estimated glomerular filtration rate (eGFR) was calculated through the Chronic Kidney Disease Epidemiology Collaboration (CKD-EPI) equation. Radiological diagnoses of pneumonia were made on the basis of the presence of at least one of the following radiographic signs on either chest X-ray or high-resolution computed tomography: mono- or bilateral consolidations, ground glass opacities, and crazy paving pattern. A calculated arterial partial pressure of oxygen (PaO_2_)/fraction of inspired oxygen ratio (PaO_2_/FiO_2_) < 300 was used to define the presence of respiratory distress. The CURB-65 score was estimated for each patient by integrating five clinical/laboratory data at admission (i.e., 1: confusion (1 point); 2: BUN >7 mmol/L (1 point); 3: respiratory rate ≥30/minute (1 point); 4: systolic blood pressure <90 mmHg or diastolic blood pressure ≤60 mmHg, as assessed using a sphygmomanometer [19] (1 point); 5: age ≥65 years (1 point)). The 4C mortality score and MuLBSTA score were also performed [20,21]. Data on the clinical course (i.e., in-hospital medical treatments and need for non-invasive ventilation (NIV)) and in-hospital outcomes (i.e., ICU admission, in-hospital death, and hospital discharge) were collected and registered in medical records as well.

### 2.3. Assessment of Brachial Artery Flow-Mediated Dilation

At the time of enrollment, bFMD was assessed by one trained ultrasonographer of the study staff. After 10 min of rest in the supine position, bFMD measurement was performed for each patient on the non-dominant arm. A linear multifrequency 5 to 12 MHz transducer (HDI 3500, Advanced Technology Laboratories, Cherry Hill, NJ, USA) was used to scan the brachial artery longitudinally just above the antecubital crease. At the R wave of the electrocardiogram, the diameter of the brachial artery was measured on the frozen image at the interface between the media and adventitia of the anterior and posterior wall. The basal measurement was performed before the inflation of a pneumatic cuff at 230–250 mmHg for 4 min on the most proximal portion of the forearm, whereas the post-hyperemic measurement was performed after its sudden deflation. All measurements were performed by the same investigator, who was blinded to the participants’ clinical data. The average of 3 measurements of the basal and post-hyperemic diameters of the brachial artery was used for statistical analysis; bFMD was calculated as 100 × [(post-hyperemia diameter − basal diameter)/basal diameter] [22,23]. The intra-observer between-occasion reproducibility of bFMD in our laboratory was 1.0 ± 1.5%.

### 2.4. Statistical Analysis

The SPSS statistical package version 24.0 (SPSS Inc, Chicago, IL, USA), was used for all statistical analyses. The Shapiro test was used to verify the normality of the study variables. Categorical variables were expressed as percentages, while continuous variables were expressed as means ± standard deviation (SD) or medians (25–75 percentile). The independent samples *t*-test, Mann–Whitney U-test, and chi-squared test were used for two-group comparisons. The Kruskal–Wallis test was used for the non-parametric comparison of multiple groups. Correlation analyses between the study variables were performed using Pearson’s and Spearman’s coefficients of correlation. To assess the risk of the composite endpoint of ICU admission/in-hospital death according to low versus high bFMD (bFMD < the median value versus bFMD ≥ the median value), an unadjusted Cox regression analysis was performed. The median value of bFMD was chosen as a cutoff point, as it was the simplest and most objective, reasonable, and convenient cutoff for this study since there are no predefined and universally accepted diagnostic criteria for distinguishing between normal and low bFMD from previous studies. The association between low bFMD (bFMD < the median value) and the composite endpoint of ICU admission/in-hospital death was further evaluated through five multi-adjusted Cox regression analyses in which low bFMD (bFMD < the median value), significant covariates of either bFMD or the composite endpoint of ICU admission/in-hospital death, and other relevant clinical variables with potential confounding effects on either bFMD or the composite endpoint of ICU admission/in-hospital death were included as independent variables (the proportional hazards assumption was previously tested through the log minus log plot and the time-dependent Cox regression analysis). In all these time-to-event analyses, the composite endpoint of ICU admission/in-hospital death was selected a priori as a cumulative index of worse in-hospital prognosis to ensure the inclusion of as many unfavorable events as possible and to yield a sufficient statistical power for this pilot study. Nonetheless, exploratory multi-adjusted Cox regression analyses were also performed to assess the association between low bFMD and either ICU admission or in-hospital death as single endpoints. Statistical significance was assumed if a null hypothesis could be rejected at *p* < 0.05.

## 3. Results

### 3.1. Baseline Characteristics of the Study Population

Four hundred and eight hospitalized COVID-19 patients were consecutively enrolled. At hospital admission, fever, dyspnea, and cough were the most prevalent clinical manifestations (70%, 62%, and 42% of patients, respectively). According to the National Institutes of Health classification of COVID-19 severity, 46 (11%), 64 (16%), and 298 (73%) patients had mild (i.e., signs and symptoms of COVID-19 without shortness of breath, dyspnea, or abnormal chest imaging), moderate (i.e., lower respiratory disease during clinical assessment or imaging and SpO_2_ ≥ 94% in room air at sea level), and severe COVID-19 (i.e., SpO_2_ < 94% in room air at sea level, PaO_2_/FiO_2_ < 300 mmHg, respiratory frequency >30 breaths/min, or lung infiltrates >50%), respectively [24]. The baseline demographic, anthropometric, clinical, and laboratory features of the study population are shown in Table 1. The median bFMD value differed significantly across the spectrum of COVID-19 severity (6.5 (3–9.5), 4.9 (3.3–7.3), and 4.1 (2.5–6) in patients with mild, moderate, and severe COVID-19, respectively, *p* for trend = 0.001).

### 3.2. Clinical Course and In-Hospital Outcomes

Clinical management of admitted patients was conducted according to available scientific evidence and recommendations at the time of enrollment. Upon hospital admission, respiratory distress was found in 298 (73%) patients, and radiographic signs of pneumonia were documented in 343 (84%) patients. Corticosteroid treatment (dexamethasone 6 mg daily) was administered to 359 (88%) patients, while antiviral therapy with remdesivir (200 mg on day 1 and 100 mg daily from day 2 to day 5) was prescribed to 135 (33%) patients, fulfilling the prescription criteria of the Italian drug agency (AIFA). Anticoagulant therapy was introduced in 369 patients (90%) (293 patients (72%) started thromboembolism prophylaxis with low-molecular-weight heparin (LMWH), while 76 patients (18%) started full anticoagulant therapy with either LMWH, vitamin K antagonists (VKAs), or direct oral anticoagulants (DOACs), depending on underlying medical conditions requiring anticoagulation and concomitant diseases). Antibiotic therapy was performed in 354 patients (87%). Vasopressor drugs were administered to 16 patients (4%).

During the hospital stay, 147 patients (36%) needed NIV, 42 patients (10%) were admitted to ICU, 76 patients (19%) died, and 118 patients (29%) met the composite endpoint of ICU admission/in-hospital death. The median time from enrollment to ICU admission was 3 (2–6) days, while the median time from enrollment to death was 9 (6–16) days.

### 3.3. Covariates of bFMD

Values of bFMD did not differ significantly according to gender, hypertension, history of previous cardiovascular (CV) event, atrial fibrillation (AF), previous venous thromboembolism (VTE), chronic obstructive pulmonary disease (COPD), current smoking, chronic kidney disease (CKD), or active cancer (*p* > 0.05 for all comparisons). In addition, no significant differences were found in bFMD values according to treatment with antiplatelet therapy, VKAs, LMWH, DOACs, oral hypoglycemic agents, statins, angiotensin-converting enzyme (ACE) inhibitors, diuretics, or calcium channel blockers (CCBs) (*p* > 0.05 for all comparisons). Instead, significantly lower bFMD values emerged in patients with type 2 diabetes compared with those without type 2 diabetes (*p* = 0.005), as well as in those who were treated with either insulin, beta blockers (BBs), or angiotensin receptor blockers (ARBs) compared with those who were not (*p* = 0.023, *p* = 0.046, and *p* = 0.006, respectively). Additionally, significantly lower bFMD values were found in patients who had radiographic signs of pneumonia, respiratory distress, or the need for NIV during hospital stay compared with those who did not (*p* < 0.001, *p* = 0.001, and *p* < 0.001, respectively). The demographic, anthropometric, clinical, and laboratory features of the study population at baseline according to low bFMD (bFMD < 4.4%, the median value) versus high bFMD (bFMD ≥ 4.4%, the median value) are summarized in Table 2.

### 3.4. Covariates of ICU Admission/In-Hospital Death

The demographic, anthropometric, clinical, and laboratory features of the study population at baseline according to the composite endpoint of ICU admission/in-hospital death are summarized in Table 3.

### 3.5. Association between Low bFMD and the Composite Endpoint of ICU Admission/In-Hospital Death

**Figure 1:**
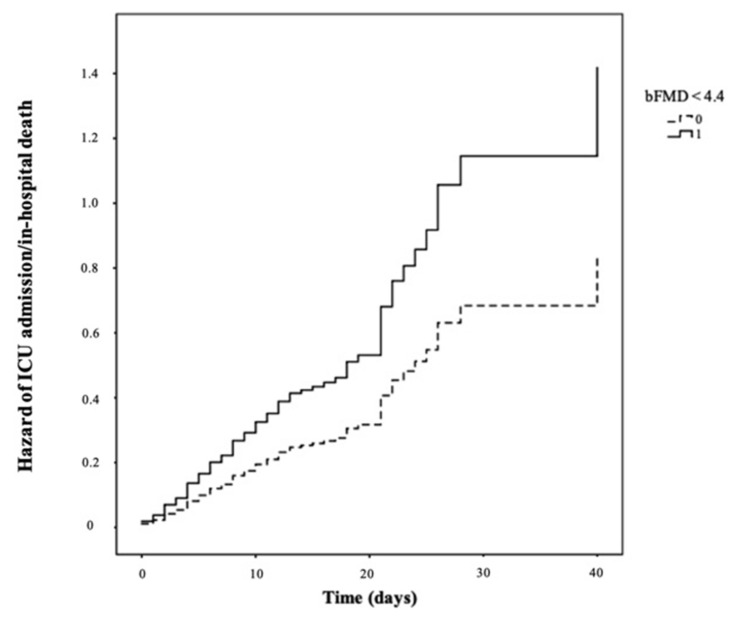
Hazard of ICU admission/in-hospital death according to low versus high bFMD (bFMD < 4.4% versus bFMD ≥ 4.4%) at hospital admission.

Significantly lower bFMD values emerged in COVID-19 patients who were either admitted to ICU or died (*p* < 0.001). The unadjusted Cox regression analysis revealed a significantly higher risk of ICU admission/in-hospital death in patients who had low bFMD (bFMD < 4.4%, the median value) compared with those who had high bFMD (bFMD ≥ 4.4%, the median value) (HR 1.675, 95% CI 1.155–2.428, *p* = 0.007) (Figure 1).

**Figure 2:**
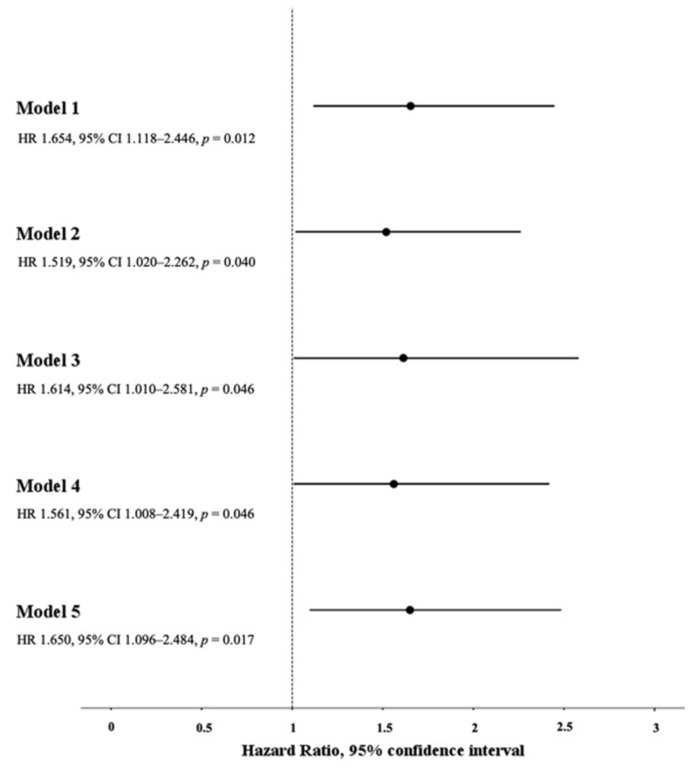
Association between low bFMD and the composite endpoint of ICU admission/in-hospital death in five Cox proportional hazard models (i.e., Models 1–5).

Five models of multi-adjusted Cox regression analysis were plotted with the time to ICU admission/in-hospital death as the time variable, ICU admission/in-hospital death as the status variable, and the following independent variables: (1) low bFMD (bFMD < 4.4%) and the main demographic and anthropometric characteristics of the study population (i.e., age, male gender, and BMI) (Model 1); (2) low bFMD (bFMD < 4.4%), the main demographic and anthropometric characteristics of the study population (i.e., age, male gender, and BMI) and the main clinical parameters of COVID-19 severity (i.e., PaO_2_/FiO_2_ and CURB-65 score) (Model 2); (3) low bFMD (bFMD < 4.4%), the main demographic and anthropometric characteristics of the study population (i.e., age, male gender, and BMI) and all significant clinical and laboratory parameters of COVID-19 severity (i.e., PaO_2_/FiO_2_, CURB-65 score, eGFR, leukocytes, CRP, D-dimer, hs-cTn, and LDH) (Model 3); (4) low bFMD (bFMD < 4.4%), the main demographic and anthropometric characteristics of the study population (i.e., age, male gender, and BMI), and CV risk factors (i.e., type 2 diabetes, current smoking, CKD, hypertension, and history of previous CV event) (Model 4); and (5) low bFMD (bFMD < 4.4%), the main demographic and anthropometric characteristics of the study population (i.e., age, male gender, and BMI), and concomitant medications at hospital admission (i.e., ACE-inhibitors, ARBs, statins, DOACs, VKAs, LMWH, anti-platelets, BBs, CCBs, diuretics, insulin, and oral hypoglycemic agents) (Model 5). Low bFMD (bFMD < 4.4%) was an independent predictor of ICU admission/in-hospital death in all five multi-adjusted Cox regression analysis models (Figure 2). In Model 2 and Model 3, low bFMD (bFMD < 4.4%) remained an independent predictor of ICU admission/in-hospital death after replacing the CURB-65 score with either the 4C mortality score or the MuLBSTA score (HR 1.610, 95% CI 1.078–2.404, *p* = 0.020 and HR 1.631, 95% CI 1.015–2.620, *p* = 0.043 for Model 2 and Model 3, respectively, including 4C mortality score instead of CURB-65 score; HR 1.499, 95% CI 1.007–2.232, *p* = 0.046 and HR 1.631, 95% CI 1.018–2.613, *p* = 0.042 for Model 2 and Model 3, respectively, including the MuLBSTA score instead of the CURB-65 score). An additional multi-adjusted Cox regression analysis model was plotted, with the time to ICU admission/in-hospital death as the time variable, ICU admission/in-hospital death as the status variable, and the following independent variables: low bFMD (bFMD < 4.4%), the main demographic and anthropometric characteristics of the study population (i.e., age, male gender, and BMI), and the main medical therapies performed during the hospital stay (i.e., corticosteroids, remdesivir, anticoagulants, antibiotics, and vasopressor drugs). In this model, low bFMD (bFMD < 4.4%) remained an independent predictor of ICU admission/in-hospital death (HR 1.658, 95% CI 1.118–2.459, *p* = 0.012).

### 3.6. Exploratory Analyses on the Association between Low bFMD and either ICU Admission or In-Hospital Death

A significant association emerged between low bFMD (bFMD < 4.4%) and ICU admission as a single endpoint in two of the five multi-adjusted Cox regression models (Supplementary Table S1). In addition, a significant association emerged between low bFMD (bFMD < 4.4%) and in-hospital death as a single endpoint in two of the five multi-adjusted Cox regression models (Supplementary Table S2).

## 4. Discussion

To our knowledge, this is the largest study extensively investigating the association between bFMD, a potential clinical and non-invasive measure of endothelial function, and in-hospital outcomes of COVID-19 patients. Indeed, in some recent studies in which bFMD was evaluated in patients with recent or ongoing COVID-19, the association between bFMD and the in-hospital prognosis of enrolled patients was not assessed [25,26,27]. In addition, a recent observational study involving 98 patients with confirmed COVID-19 investigated only the prospective association between bFMD and in-hospital mortality [28].

Three main results of the present study deserve discussion: (1) lower bFMD values at hospital admission were found in patients who had more severe clinical manifestations of COVID-19 (i.e., radiological diagnosis of pneumonia, respiratory distress, and need for NIV); (2) the risk of the composite endpoint of ICU admission/in-hospital death was significantly higher in patients with bFMD < 4.4% compared with those with bFMD ≥ 4.4%; and (3) the prospective association between low bFMD (bFMD < 4.4%) and unfavorable in-hospital prognosis of COVID-19 was independent of the indices of clinical severity at hospital admission, pre-existing medical conditions/therapies, and in-hospital therapies.

The finding of reduced bFMD values in patients with more severe COVID-19 manifestations is consistent with the results of previous cross-sectional, retrospective, and prospective studies, which have shown a significant and direct association between COVID-19 severity and different direct/indirect measures of endothelial function other than bFMD [15,16,17,18,29]. In addition, this finding might support two different albeit not mutually exclusive pathophysiological scenarios in which severe COVID-19 may be either the trigger (i.e., causality) or the consequence (i.e., reverse causality) of endothelial dysfunction. From a biological perspective, the plausibility of the first assumption (causality) relies on several lines of evidence. First, SARS-CoV-2 can directly infect endothelial cells, and SARS-CoV-2 viral load has been reported to be closely associated with the entities of both endothelial damage and clinical severity in COVID-19 patients [30,31,32]. Second, the overproduction of proinflammatory cytokines that may be induced by SARS-CoV-2 infection, the so-called cytokine storm, is able to promote endothelial damage and has been reported to display a close link with disease severity [33,34]. Third, other viral infections (i.e., HIV infection and influenza infection) have been previously reported to impair endothelial function in their high viral replication state and acute inflammatory state [35,36]. However, reverse causality is also plausible when examining the association between COVID-19 severity and the degree of endothelial dysfunction. In this regard, there is evidence that by altering vascular barrier integrity, promoting a pro-coagulative state and microthrombus formation, and enhancing inflammatory cell infiltration, endothelial injury has a crucial pathogenic role in the onset of both acute respiratory distress syndrome and multiple organ failure in COVID-19 patients [37]. In addition, it has been previously reported that nitric oxide (NO), a well-known endothelial mediator, has significant anti-inflammatory and immune-modulating activity and can exert viricidal effects against a wide range of viruses, including coronaviruses [38,39]. Therefore, NO depletion due to endothelial injury may promote pathogenic mechanisms of COVID-19. Moreover, the use of drugs that increase NO availability (e.g., ACE inhibitors) has been associated with favorable COVID-19 outcomes, whereas treatment with drugs inhibiting NO production/release (e.g., proton pump inhibitors) has been associated with worse COVID-19 prognosis [40,41,42,43]. Accordingly, COVID-19 has been defined as an endothelial disease in which the spectrum of clinical severity varies according to the entity of endothelial damage [32]. Nonetheless, potential confounding factors of the pathophysiological association between endothelial injury and COVID-19 severity should be considered as well. Indeed, COVID-19 patients are more likely to present underlying conditions such as advanced age, hypertension, diabetes, and CV diseases, which are associated with both endothelial dysfunction and progression to severe clinical manifestations of COVID-19 [44].

Regardless of these speculative hypotheses on the direction of the association between the severity of COVID-19 manifestations and endothelial dysfunction, we found that low bFMD predicted an unfavorable prognosis independently of multiple confounders. The latter finding, beyond adding further support for a possible pathophysiological link between endothelial injury and COVID-19 severity, has important clinical implications. The first is the possible utility of bFMD measurement at hospital admission to identify COVID-19 patients who are more likely to progress to the most severe clinical manifestations and worse prognosis. The second is the need to develop effective therapies aimed at restoring endothelial function to halt COVID-19 progression and improve clinical outcomes. Regarding the first assumption, it should be emphasized that bFMD measurement is a non-invasive and easy bedside procedure that provides a result in real time [45]. Therefore, the measurement of bFMD in COVID-19 patients at hospital admission might refine our prognostic ability and possibly improve decision-making about medical care intensity [14]. In this regard, as several drugs against severe COVID-19 are currently under investigation and being tested in clinical trials, the measurement of bFMD upon hospital admission may provide an option to select patients who might benefit from a more intensive treatment approach based on experimental drugs beyond the standard of care. Nonetheless, it should be acknowledged that bFMD assessment is operator-dependent, which can complicate the interpretation of the obtained results and influence further clinical decisions; this may potentially limit its widespread employment as a prognostic tool in COVID-19 clinics. Regarding the second assumption, it should be considered that given the poor availability of effective therapies targeting viral replication and immune response, the use of strategies aimed at improving endothelial function may be a valuable approach to protect vulnerable patients against unfavorable COVID-19 outcomes [12]. To this end, several chemical compounds have been proposed or are under investigation as add-on treatments for COVID-19 on the basis of their well-known endothelium-protective effects, including renin angiotensin system (RAS) inhibitors and statins [12,46,47]. However, their use is still limited because of the absence of robust evidence from intervention studies. Therefore, pharmacologic approaches aimed at restoring endothelial function in COVID-19 remain an open research area.

The limitations of the present study should be acknowledged. First, the small sample size obtained from a single hospital may limit the generalizability of the observed results. Second, an assessment of additional markers of endothelial function beyond bFMD, which could have supported the study results, was not performed. Particularly, brachial artery endothelium-independent dilation (i.e., nitroglycerine-induced vasodilation) was not measured. This could have acted as a control test to ensure that impaired vasodilatation did not occur because of the decreased reactivity of vascular smooth muscle cells to NO or alterations in vascular structure and instead occurred because of the impaired production of NO by endothelial cells. Third, a comparison of bFMD values between COVID-19 cases and non-COVID-19 controls, which could have strengthened the study results, was not possible. Fourth, the absence of a long-term follow-up for patients who were discharged alive only allowed us to assess predictors of in-hospital prognosis.

## 5. Conclusions

This study shows that low bFMD, a potential clinical and non-invasive measure of endothelial dysfunction, correlates with COVID-19 severity and predicts worse in-hospital outcomes in COVID-19 patients. Therapeutic strategies promoting endothelial protection/repair to prevent the most severe complications of COVID-19 are awaited.

## Figures and Tables

**Table 1:** Baseline characteristics of the study population.

	Total Study Population *n* = 408
**Age, years**	72 (16)
**Male gender, %**	52
**BMI, kg/m^2^**	26.5 (4.3)
**Current smoking, %**	16
**Hypertension, %**	61
**Type 2 diabetes, %**	19
**CKD, %**	11
**Previous CV event, %**	16
**Active cancer, %**	9
**Previous VTE, %**	3
**AF, %**	15
**COPD, %**	12
**ACE inhibitors, %**	27
**ARBs, %**	17
**Statins, %**	19
**DOACs, %**	10
**VKAs, %**	2
**LMWH, %**	19
**Anti-platelets, %**	23
**BBs, %**	25
**CCBs, %**	24
**Diuretics, %**	32
**Insulin, %**	13
**Oral hypoglycemic agents, %**	9
**SBP, mmHg**	131 (21)
**DBP, mmHg**	80 (11)
**Leukocytes, X 10^3^/** **μL**	7.2 (5.1–10.3)
**Platelets, X 10^3^/** **μL**	203 (154–265)
**D-dimer, ng/mL**	839 (531–1732)
**hs-cTn, ng/L**	13.5 (6.9–29.5)
**CRP, mg/dL**	6.5 (3.1–11.6)
**eGFR, mL/min**	71 (27)
**LDH, UI/L**	292 (224–407)
**PaO_2_/FiO_2_**	250 (171–304)
**CURB-65 score**	2 (1–3)
**4C mortality score**	12 (9–15)
**MuLBSTA score**	8 (4–11)
**bFMD, %**	4.4 (2.7–6.8)

Values are expressed as means (SD), medians (25–75 percentile), or percentages. Abbreviations: ACE, angiotensin-converting enzyme; AF, atrial fibrillation; ARBs, angiotensin receptor blockers; BBs, beta-blockers; bFMD, brachial flow-mediated dilation; BMI, body mass index; CCBs, calcium channel blockers; CKD, chronic kidney disease; COPD, chronic obstructive pulmonary disease; CRP, C-reactive protein; CV, cardiovascular; DBP, diastolic blood pressure; DOACs, direct oral anticoagulants; eGFR, estimated glomerular filtration rate; FiO_2_, fraction of inspiration oxygen; hs-cTn, high-sensitivity cardiac troponin; LDH, lactate dehydrogenase; LMWH, low-molecular-weight heparin; PaO_2_, arterial partial pressure of oxygen; SBP, systolic blood pressure; VKAs, vitamin K antagonists; VTE, venous thromboembolism.

**Table 2:** Baseline characteristics of the study population according to low bFMD (bFMD < 4.4%) versus high bFMD (bFMD ≥ 4.4%).

	bFMD < 4.4% *n* = 201	bFMD ≥ 4.4% *n* = 207	*p*
**Age, years**	73 (14)	72 (18)	0.323
**Male gender, %**	54	51	0.479
**BMI, kg/m^2^**	27.1 (4.6)	26.0 (4.1)	0.022
**Current smoking, %**	19	12	0.198
**Hypertension, %**	65	57	0.097
**Type 2 diabetes, %**	24	14	0.011
**CKD, %**	13	10	0.226
**Previous CV event, %**	18	14	0.276
**Active cancer, %**	9	10	0.956
**Previous VTE, %**	2	4	0.307
**AF, %**	16	13	0.401
**COPD, %**	12	11	0.899
**ACE inhibitors, %**	28	27	0.769
**ARBs, %**	19	11	0.027
**Statins, %**	21	16	0.215
**DOACs, %**	11	9	0.435
**VKAs, %**	2	2	0.776
**LMWH, %**	17	21	0.338
**Anti-platelets, %**	26	21	0.174
**BBs, %**	34	24	0.022
**CCBs, %**	24	18	0.163
**Diuretics, %**	36	32	0.380
**Insulin, %**	15	9	0.071
**Oral hypoglycemic agents, %**	12	9	0.273
**SBP, mmHg**	131 (20)	131 (21)	0.682
**DBP, mmHg**	76 (11)	77 (11)	0.492
**Leukocytes, X 10^3^/** **μL**	9.4 (5.3–11.0)	8.1 (4.9–10.0)	0.155
**Platelets, X 10^3^/** **μL**	216 (157–257)	223 (146–269)	0.740
**D-dimer, ng/mL**	871 (539–1682)	824 (531–1765)	0.921
**hs-cTn, ng/L**	14.3 (7.1–40.5)	12.7 (6.2–26.3)	0.084
**CRP, mg/dL**	6.5 (3.2–12.4)	6.5 (3.2–12.4)	0.484
**eGFR, mL/min**	68 (26)	74 (28)	0.079
**LDH, UI/L**	305 (234–416)	278 (214–396)	0.101
**PaO_2_/FiO_2_**	242 (159–291)	266 (185–319)	0.010
**CURB-65 score**	2 (1–3)	2 (1–3)	0.253
**4C mortality score**	12 (9–15)	11 (7–14)	0.092
**MuLBSTA score**	12 (9–15)	7 (4–9)	0.014

Values are expressed as means (SD), medians (25–75 percentile), or percentages. Abbreviations: ACE, angiotensin-converting enzyme; AF, atrial fibrillation; ARBs, angiotensin receptor blockers; BBs, beta-blockers; bFMD, brachial flow-mediated dilation; BMI, body mass index; CCBs, calcium channel blockers; CKD, chronic kidney disease; COPD, chronic obstructive pulmonary disease; CRP, C-reactive protein; CV, cardiovascular; DBP, diastolic blood pressure; DOACs, direct oral anticoagulants; eGFR, estimated glomerular filtration rate; FiO_2_, fraction of inspiration oxygen; hs-cTn, high-sensitivity cardiac troponin; LDH, lactate dehydrogenase; LMWH, low-molecular-weight heparin; PaO_2_, arterial partial pressure of oxygen; SBP, systolic blood pressure; VKAs, vitamin-K antagonists; VTE, venous thromboembolism.

**Table 3:** Baseline characteristics of the study population according to the composite endpoint of ICU admission/in-hospital death.

	Non-ICU Admitted/ Discharged Alive *n* = 290	ICU Admitted/ Non-Survivors *n* = 118	*p*
**Age, years**	70 (17)	78 (13)	<0.001
**Male gender, %**	50	57	0.182
**BMI, kg/m^2^**	26.8 (4.5)	25.7 (4.1)	0.038
**Current smoking, %**	17	15	0.719
**Hypertension, %**	60	66	0.248
**Type 2 diabetes, %**	16	28	0.004
**CKD, %**	9	19	0.004
**Previous CV event, %**	14	23	0.022
**Active cancer, %**	9	11	0.506
**Previous VTE, %**	3	3	0.988
**AF, %**	13	20	0.094
**COPD, %**	12	11	0.786
**ACE inhibitors, %**	28	26	0.808
**ARBs, %**	14	18	0.382
**Statins, %**	18	19	0.809
**DOACs, %**	9	11	0.581
**VKAs, %**	2	3	0.293
**LMWH, %**	17	24	0.082
**Anti-platelets, %**	21	30	0.015
**BBs, %**	28	32	0.361
**CCBs, %**	18	28	0.026
**Diuretics, %**	32	40	0.119
**Insulin, %**	9	18	0.007
**Oral hypoglycemic agents, %**	9	14	0.157
**SBP, mmHg**	132 (20)	128 (22)	0.053
**DBP, mmHg**	78 (11)	74 (12)	0.004
**Leukocytes, X 10^3^/** **μL**	7.0 (4.9–10.2)	7.7 (5.8–11.1)	0.030
**Platelets, X 10^3^/** **μL**	208 (159–268)	192 (144–254)	0.135
**D-dimer, ng/mL**	757 (532–1538)	1211 (529–2807)	0.015
**hs-cTn, ng/L**	10.2 (5.6–23)	24.1 (11.4–44.6)	<0.001
**CRP, mg/dL**	5.7 (2.4–10.0)	9.1 (4.7–15.0)	<0.001
**eGFR, mL/min**	76 (26)	58 (27)	<0.001
**LDH, UI/L**	268 (216–368)	361 (263–466)	<0.001
**PaO_2_/FiO_2_**	271 (213–319)	159 (114–257)	<0.001
**CURB-65 score**	2 (1–2)	2 (1–3)	<0.001
**4C mortality score**	11 (7–13)	14 (12–16)	<0.001
**MuLBSTA score**	7 (4–9)	9 (6–13)	<0.001
**bFMD, %**	4.7 (3.0–7.4)	3.6 (2.0–5.2)	<0.001

Values are expressed as means (SD), medians (25–75 percentile), or percentages. Abbreviations: ACE, angiotensin-converting enzyme; AF, atrial fibrillation; ARBs, angiotensin receptor blockers; BBs, beta-blockers; bFMD, brachial flow-mediated dilation; BMI, body mass index; CCBs, calcium channel blockers; CKD, chronic kidney disease; COPD, chronic obstructive pulmonary disease; CRP, C-reactive protein; CV, cardiovascular; DBP, diastolic blood pressure; DOACs, direct oral anticoagulants; eGFR, estimated glomerular filtration rate; FiO_2_, fraction of inspiration oxygen; hs-cTn, high-sensitivity cardiac troponin; ICU, intensive care unit; LDH, lactate dehydrogenase; LMWH, low-molecular-weight heparin; PaO_2_, arterial partial pressure of oxygen; SBP, systolic blood pressure; VKAs, vitamin K antagonists; VTE, venous thromboembolism.

## Data Availability

The data presented in this study are available on request from the corresponding author.

## Supplementary Materials

The following are available online at https://www.mdpi.com/article/10.3390/jcm10225456/s1, Table S1: Association between low bFMD and ICU admission, Table S2: Association between low bFMD and in-hospital deaths.

## References

[1] WHO Statement on the Sixth Meeting of the International Health Regulations (2005) Emergency Committee regarding the Coronavirus Disease (COVID-19) Pandemic. https://www.who.int.

[2] WHO Situation by Country, Territory & Area. https://covid19.who.int.

[3] Asch D.A., Sheils N.E., Islam M.N., Chen Y., Werner R.M., Buresh J., Doshi J.A. (2021). Variation in US Hospital Mortality Rates for Patients Admitted With COVID-19 During the First 6 Months of the Pandemic. JAMA Intern. Med..

[4] Ciceri F., Ruggeri A., Lembo R., Puglisi R., Landoni G., Zangrillo A., COVID-BioB Study Group (2020). Decreased in-hospital mortality in patients with COVID-19 pneumonia. Pathog. Glob. Health.

[5] Bose S., Adapa S., Aeddula N.R., Roy S., Nandikanti D., Vupadhyayula P.M., Naramala S., Gayam V., Muppidi V., Konala V.M. (2020). Medical Management of COVID-19: Evidence and Experience. J. Clin. Med. Res..

[6] CDC Information for Clinicians on Investigational Therapeutics for Patients with COVID-19. https://www.cdc.gov.

[7] Bianconi V., Violi F., Fallarino F., Pignatelli P., Sahebkar A., Pirro M. (2020). Is Acetylsalicylic Acid a Safe and Potentially Useful Choice for Adult Patients with COVID-19?. Drugs.

[8] Shojaei A., Salari P. (2020). COVID-19 and off label use of drugs: An ethical viewpoint. Daru.

[9] Wynants L., Sotgiu G. (2021). Improving clinical management of COVID-19: The role of prediction models. Lancet Respir. Med..

[10] Nägele M.P., Haubner B., Tanner F.C., Ruschitzka F., Flammer A.J. (2020). Endothelial dysfunction in COVID-19: Current findings and therapeutic implications. Atherosclerosis.

[11] Bernard I., Limonta D., Mahal L.K., Hobman T.C. (2020). Endothelium Infection and Dysregulation by SARS-CoV-2: Evidence and Caveats in COVID-19. Viruses.

[12] Evans P.C., Rainger G.E., Mason J.C., Guzik T.J., Osto E., Stamataki Z., Neil D., Hoefer I.E., Fragiadaki M., Waltenberger J. (2020). Endothelial dysfunction in COVID-19: A position paper of the ESC Working Group for Atherosclerosis and Vascular Biology, and the ESC Council of Basic Cardiovascular Science. Cardiovasc. Res..

[13] Chang R., Mamun A., Dominic A., Le N.T. (2021). SARS-CoV-2 Mediated Endothelial Dysfunction: The Potential Role of Chronic Oxidative Stress. Front. Physiol..

[14] Yoganandamoorthy S., Munasinghe M.A.D.S.N., Wanigasuriya L.V.U., Priyankara M.K.K., Jayasinghe S. (2020). Non-invasive assessment of endothelial dysfunction: A novel method to predict severe COVID-19?. Med. Hypotheses.

[15] Guervilly C., Burtey S., Sabatier F., Cauchois R., Lano G., Abdili E., Daviet F., Arnaud L., Brunet P., Hraiech S. (2020). Circulating Endothelial Cells as a Marker of Endothelial Injury in Severe COVID-19. J. Infect. Dis..

[16] Falcinelli E., Petito E., Becattini C., De Robertis E., Paliani U., Sebastiano M., Vaudo G., Guglielmini G., Paciullo F., Cerotto V. (2021). COVIR study investigators. Role of endothelial dysfunction in the thrombotic complications of COVID-19 patients. J. Infect..

[17] Goshua G., Pine A.B., Meizlish M.L., Chang C.H., Zhang H., Bahel P., Baluha A., Bar N., Bona R.D., Burns A.J. (2020). Endotheliopathy in COVID-19-associated coagulopathy: Evidence from a single-centre, cross-sectional study. Lancet Haematol..

[18] Philippe A., Chocron R., Gendron N., Bory O., Beauvais A., Peron N., Khider L., Guerin C.L., Goudot G., Levasseur F. (2021). Circulating Von Willebrand factor and high molecular weight multimers as markers of endothelial injury predict COVID-19 in-hospital mortality. Angiogenesis.

[19] Schillaci G., Mannarino M.R., Pucci G., Pirro M., Helou J., Savarese G., Vaudo G., Mannarino E. (2007). Age-specific relationship of aortic pulse wave velocity with left ventricular geometry and function in hypertension. Hypertension.

[20] Knight S.R., Ho A., Pius R., Buchan I., Carson G., Drake T.M., Dunning J., Fairfield C.J., Gamble C., Green C.A. (2020). Risk stratification of patients admitted to hospital with covid-19 using the ISARIC WHO Clinical Characterisation Protocol: Development and validation of the 4C Mortality Score. BMJ.

[21] Guo L., Wei D., Zhang X., Wu Y., Li Q., Zhou M., Qu J. (2019). Clinical Features Predicting Mortality Risk in Patients With Viral Pneumonia: The MuLBSTA Score. Front. Microbiol..

[22] Pirro M., Mannarino M.R., Francisci D., Schiaroli E., Bianconi V., Bagaglia F., Sahebkar A., Mannarino E., Baldelli F. (2016). Urinary albumin-to-creatinine ratio is associated with endothelial dysfunction in HIV-infected patients receiving antiretroviral therapy. Sci. Rep..

[23] Pasqualini L., Cortese C., Marchesi S., Siepi D., Pirro M., Vaudo G., Liberatoscioli L., Gnasso A., Schillaci G., Mannarino E. (2005). Paraoxonase-1 activity modulates endothelial function in patients with peripheral arterial disease. Atherosclerosis.

[24] NIH COVID-19 Treatment Guidelines. https://www.covid19treatmentguidelines.nih.gov.

[25] Ratchford S.M., Stickford J.L., Province V.M., Stute N., Augenreich M.A., Koontz L.K., Bobo L.K., Stickford A.S.L. (2021). Vascular alterations among young adults with SARS-CoV-2. Am. J. Physiol. Heart Circ. Physiol..

[26] Kalinskaya A., Dukhin O., Molodtsov I., Maltseva A., Sokorev D., Elizarova A., Sapozhnikova O., Glebova K., Stonogina D., Shakhidzhanov S. (2020). Dynamics of coagulopathy in patients with different COVID-19 severity. medRxiv.

[27] Heubel A.D., Viana A.A., Linares S.N., do Amaral V.T., Schafauser N.S., de Oliveira G.Y.O., Ramírez P.C., Martinelli B., da Silva Alexandre T., Borghi Silva A. (2021). Determinants of endothelial dysfunction in non-critically ill hospitalized COVID-19 patients: A cross-sectional study. Obesity.

[28] Oliveira M.R., Back G.D., da Luz Goulart C., Domingos B.C., Arena R., Borghi-Silva A. (2021). Endothelial function provides early prognostic information in patients with COVID-19: A cohort study. Respir. Med..

[29] Sabioni L., De Lorenzo A., Lamas C., Muccillo F., Castro-Faria-Neto H.C., Estato V., Tibirica E. (2021). Systemic microvascular endothelial dysfunction and disease severity in COVID-19 patients: Evaluation by laser Doppler perfusion monitoring and cytokine/chemokine analysis. Microvasc. Res..

[30] Libby P., Lüscher T. (2020). COVID-19 is, in the end, an endothelial disease. Eur. Heart J..

[31] Varga Z., Flammer A.J., Steiger P., Haberecker M., Andermatt R., Zinkernagel A.S., Mehra M.R., Schuepbach R.A., Ruschitzka F., Moch H. (2020). Endothelial cell infection and endotheliitis in COVID-19. Lancet.

[32] Fajnzylber J., Regan J., Coxen K., Corry H., Wong C., Rosenthal A., Worrall D., Giguel F., Piechocka-Trocha A., Atyeo C. (2020). Massachusetts Consortium for Pathogen Readiness. SARS-CoV-2 viral load is associated with increased disease severity and mortality. Nat. Commun..

[33] Pearce L., Davidson S.M., Yellon D.M. (2020). The cytokine storm of COVID-19: A spotlight on prevention and protection. Expert. Opin. Ther. Targets.

[34] Zhao Z., Wei Y., Tao C. (2021). An enlightening role for cytokine storm in coronavirus infection. Clin. Immunol..

[35] Solages A., Vita J.A., Thornton D.J., Murray J., Heeren T., Craven D.E., Horsburgh C.R. (2006). Endothelial function in HIV-infected persons. Clin. Infect. Dis..

[36] Marchesi S., Lupattelli G., Lombardini R., Sensini A., Siepi D., Mannarino M., Vaudo G., Mannarino E. (2005). Acute inflammatory state during influenza infection and endothelial function. Atherosclerosis.

[37] Teuwen L.A., Geldhof V., Pasut A., Carmeliet P. (2020). COVID-19: The vasculature unleashed. Nat. Rev. Immunol..

[38] Keyaerts E., Vijgen L., Chen L., Maes P., Hedenstierna G., Van Ranst M. (2004). Inhibition of SARS-coronavirus infection in vitro by S-nitroso-N-acetylpenicillamine, a nitric oxide donor compound. Int. J. Infect. Dis..

[39] Fiorucci S., Mencarelli A., Distrutti E., Baldoni M., del Soldato P., Morelli A. (2004). Nitric oxide regulates immune cell bioenergetic: A mechanism to understand immunomodulatory functions of nitric oxide-releasing anti-inflammatory drugs. J. Immunol..

[40] Lee S.W., Ha E.K., Yeniova A.Ö., Moon S.Y., Kim S.Y., Koh H.Y., Yang J.M., Jeong S.J., Moon S.J., Cho J.Y. (2021). Severe clinical outcomes of COVID-19 associated with proton pump inhibitors: A nationwide cohort study with propensity score matching. Gut.

[41] Chen W.T., Shie C.B., Yang C.C., Lee T.M. (2019). Blockade of Cardiac Proton Pump Impairs Ventricular Remodeling Through a Superoxide-DDAH-Dependent Pathway in Infarcted Rats. Acta Cardiol. Sin..

[42] Ancion A., Tridetti J., Trung M.L.N., Oury C., Lancellotti P. (2019). A Review of the Role of Bradykinin and Nitric Oxide in the Cardioprotective Action of Angiotensin-Converting Enzyme Inhibitors: Focus on Perindopril. Cardiol. Ther..

[43] Reynolds H.R., Adhikari S., Iturrate E. (2020). RAAS Inhibitors and Risk of Covid-19. Reply. N. Engl. J. Med..

[44] Bermejo-Martin J.F., Almansa R., Torres A., González-Rivera M., Kelvin D.J. (2020). COVID-19 as a cardiovascular disease: The potential role of chronic endothelial dysfunction. Cardiovasc. Res..

[45] Alley H., Owens C.D., Gasper W.J., Grenon S.M. (2014). Ultrasound assessment of endothelial-dependent flow-mediated vasodilation of the brachial artery in clinical research. J. Vis. Exp..

[46] Ganjali S., Bianconi V., Penson P.E., Pirro M., Banach M., Watts G.F., Sahebkar A. (2020). Commentary: Statins, COVID-19, and coronary artery disease: Killing two birds with one stone. Metabolism.

[47] Momtazi-Borojeni A.A., Banach M., Reiner Ž., Pirro M., Bianconi V., Al-Rasadi K., Sahebkar A. (2021). Interaction Between Coronavirus S-Protein and Human ACE2: Hints for Exploring Efficient Therapeutic Targets to Treat COVID-19. Angiology.

