# Supplementary material for: Low Brachial Artery Flow-Mediated Dilation Predicts Worse Prognosis in Hospitalized Patients with COVID-19
Source: J Clin Med. 2021 Nov 22;10(22):5456. doi: 10.3390/jcm10225456 (PMC8621380; doi:10.3390/jcm10225456)
Supplement: Supplementary file 1 [file jcm-10-05456-s001.zip › jcm-1427705-supplementary.pdf]

**Table S1.** Association between low bFMD and ICU admission.

| **Model 1** | **HR** | **95% CI** | ***p*** |
|---|---|---|---|
| Low bFMD (<4.4%) | 2.258 | 1.088–4.684 | 0.029 |
| **Model 2** | **HR** | **95% CI** | ***p*** |
| Low bFMD (<4.4%) | 1.670 | 0.795–3.507 | 0.176 |
| **Model 3** | **HR** | **95% CI** | ***p*** |
| Low bFMD (<4.4%) | 1.905 | 0.817–4.445 | 0.136 |
| **Model 4** | **HR** | **95% CI** | ***p*** |
| Low bFMD (<4.4%) | 3.113 | 1.314–7.373 | 0.010 |
| **Model 5** | **HR** | **95% CI** | ***p*** |
| Low bFMD (<4.4%) | 1.886 | 0.871–4.083 | 0.107 |

Five Cox proportional hazard models (i.e., Model 1–5) were plotted by including the time to ICU admission/in-hospital death as the time variable, the composite endpoint of ICU-admission/in-hospital death as the status variable and the following covariates: 1) low bFMD (bFMD < 4.4%), age, male gender, and BMI (Model 1), (2) low bFMD (bFMD < 4.4%), age, male gender, BMI, $PaO_2/FiO_2$ and CURB-65 score (Model 2), (3) low bFMD (bFMD < 4.4%), age, male gender, BMI, $PaO_2/FiO_2$, CURB-65 score, eGFR, leukocytes, CRP, D-dimer, hs-cTn, and LDH (Model 3), (4) low bFMD (bFMD < 4.4%), age, male gender, BMI, type 2 diabetes, current smoking, CKD, hypertension, and history of previous CV event (Model 4), (5) low bFMD (bFMD < 4.4%, age, male gender, BMI, ACE-inhibitors, ARBs, statins, DOACs, VKAs, LMWH, anti-platelets, BBs, CCBs, diuretics, insulin, and oral hypoglycemic agents (Model 5). Abbreviations: bFMD, brachial flow-mediated dilation; CI, confidence interval; HR, hazard ratio.

**Table S2.** Association between low bFMD and in-hospital deaths.

| **Model 1** | **HR** | **95% CI** | ***p*** |
|---|---|---|---|
| Low bFMD (<4.4%) | 1.533 | 1.004–2.343 | 0.048 |
| **Model 2** | **HR** | **95% CI** | ***p*** |
| Low bFMD (<4.4%) | 1.402 | 0.911–2.157 | 0.124 |
| **Model 3** | **HR** | **95% CI** | ***p*** |
| Low bFMD (<4.4%) | 1.427 | 0.846–2.408 | 0.182 |
| **Model 4** | **HR** | **95% CI** | ***p*** |
| Low bFMD (<4.4%) | 1.325 | 0.824–2.132 | 0.246 |
| **Model 5** | **HR** | **95% CI** | ***p*** |
| Low bFMD (<4.4%) | 1.654 | 1.054–2.594 | 0.029 |

Five Cox proportional hazard models (i.e., Model 1–5) were plotted by including the time to ICU admission/in-hospital death as the time variable, the composite endpoint of ICU-admission/in-hospital death as the status variable and the following covariates: (1) low bFMD (bFMD < 4.4%), age, male gender, and BMI (Model 1), (2) low bFMD (bFMD < 4.4%), age, male gender, BMI, $PaO_2/FiO_2$ and CURB-65 score (Model 2), (3) low bFMD (bFMD < 4.4%), age, male gender, BMI, $PaO_2/FiO_2$, CURB-65 score, eGFR, leukocytes, CRP, D-dimer, hs-cTn, and LDH (Model 3), (4) low bFMD (bFMD < 4.4%), age, male gender, BMI, type 2 diabetes, current smoking, CKD, hypertension, and history of previous CV event (Model 4), (5) low bFMD (bFMD < 4.4%, age, male gender, BMI, ACE-inhibitors, ARBs, statins, DOACs, VKAs, LMWH, anti-platelets, BBs, CCBs, diuretics, insulin, and oral hypoglycemic agents (Model 5). Abbreviations: bFMD, brachial flow-mediated dilation; CI, confidence interval; HR, hazard ratio.
